# Neuro-ophthalmic observation and 16-month follow-up of horner syndrome after thyroidectomy: A case report

**DOI:** 10.1097/MD.0000000000047236

**Published:** 2026-01-16

**Authors:** Xiao-Ming Li, Bo Yang, Song Yang, Bo Wang, Shan-Xue Liu

**Affiliations:** a Department of Ophthalmology, The Affiliated Hospital of ChangchunUniversity of Chinese Medicine, Changchun, Jilin Province, China; b Department of Medical Retina, The Second Hospital of Jilin University, Changchun, Jilin, China.

**Keywords:** anisocoria, anisocoriathyroidectomy, brimonidine tartrate, Horner syndrome

## Abstract

**Rationale:**

Horner syndrome (HS) is a rare but underrecognized complication of thyroidectomy, typically resulting from intraoperative injury to the cervical sympathetic chain. Its clinical manifestations include ptosis, miosis, enophthalmos, anhidrosis, and vasodilation. However, data on causative factors, progression of ocular signs, and clinical recovery timing remain limited. This study aimed to clarify these aspects to establish reference intervals for patient management.

**Patient concerns:**

A 29-year-old female developed postoperative miosis and ptosis following endoscopic thyroidectomy. Her primary concern was persistent ocular symptoms affecting daily life.

**Diagnoses:**

HS was confirmed through comprehensive evaluation, including ptosis measurement, scotopic pupillometry, and positive response to 0.2% brimonidine testing.

**Interventions:**

She received intravenous methylprednisolone (80 mg/day for 10 days) and oral methylcobalamin (0.5 mg 3 times daily for 30 days).

**Outcomes:**

At 16-month follow-up, partial persistence of miosis and ptosis was observed; however, facial sweating normalized, and other ocular symptoms resolved completely.

**Lessons:**

HS should be considered a potential complication of thyroidectomy, even when rare. Simple clinical and pharmacologic tests (e.g., brimonidine test) suffice for confirmation. Notably, complete recovery from iatrogenic HS secondary to endoscopic thyroidectomy may exceed 12 months, highlighting the need for long-term follow-up and patient counseling.

## 1. Introduction

Horner syndrome (HS) is characterized by the classic triad of miosis, ptosis, and enophthalmos due to oculo-sympathetic pathway disruption.^[1,2]^ The incidence of HS resulting from endoscopic thyroid surgery ranges from 0.03% to 0.48%.^[3]^ In a cohort of 2611 patients who underwent thyroid surgery, the incidence of postoperative HS was 0.39% in the endoscopic group (n = 513) and 0.29% in the open surgery group (n = 2098).^[4]^ This risk stems from the variable and intimate anatomical relationship between the thyroid gland and the cervical sympathetic chain. Despite advances in surgical techniques, avoiding this complication remains challenging.

Previous case reports have primarily concentrated on descriptions of surgical procedures and associated ocular symptoms, often lacking clear ocular photographs, particularly pupillary images, and documentation of pharmacological testing for HS. Notably, there is a deficiency in standardized documentation, which includes ocular photographs obtained under darkroom conditions, clearly depicting both pupils and demonstrating relative anisocoria; pharmacological testing results confirming HS; and longitudinal follow-up documentation, including clear ocular photographs and bilateral pupillary measurements, extending beyond 1 year postoperatively. We present a case of a patient who developed left miosis, ptosis, and left-sided anhidrosis following endoscopic thyroidectomy for thyroid carcinoma. The diagnosis was confirmed through detailed neuro-ophthalmic evaluation and a 0.2% brimonidine tartrate challenge test. Notably, complete resolution of ocular manifestations was observed at the 16-month follow-up.

## 2. Case report

The study was approved by the Ethics Committee of the Affiliated Hospital of Changchun University of Chinese Medicine (Approval No. [CCZYFYLL-SQ-2025]). The patient provided consent for the case to be published.

A 29-year-old female patient presented with persistent left upper eyelid ptosis for 3 days following radical thyroidectomy for thyroid carcinoma. Two years earlier, a routine examination revealed an asymptomatic thyroid nodule. Subsequent fine-needle aspiration cytology of the nodule at the left inferior pole demonstrated atypical cells suggestive of papillary thyroid carcinoma, and molecular testing confirmed the BRAF V600E mutation. Preoperative thyroid and neck ultrasonography revealed the following findings: a normal-sized thyroid gland exhibiting heterogeneous parenchymal echogenicity; a 0.38 × 0.31-cm irregular, markedly hypoechoic solid nodule located at the left inferior pole near the isthmus, without the presence of microcalcifications; unremarkable vascularity observed on colour Doppler flow imaging (CDFI), with normal flow velocity; and multiple oval-shaped lymph nodes in level VI, the largest measuring 0.93 × 0.28 cm, demonstrating preserved architecture and absent vascular flow on CDFI. Thyroid ultrasound revealed a markedly hypoechoic nodule in the left lobe, which was classified as ACR TI-RADS. Based on a comprehensive evaluation, the preoperative clinical diagnosis was malignant thyroid neoplasm. On December 26, 2023, the patient underwent endoscopic extended radical resection for left thyroid lobe carcinoma under general anesthesia, which included left thyroid lobectomy with isthmusectomy, central compartment lymph node dissection, and recurrent laryngeal nerve exploration. The nerve monitoring system was used intraoperatively, and laparoscopic access was established. An ultrasonic scalpel was used to dissect the linea alba cervicalis and the infrahyoid muscles. The thyroid lobes were exposed by dissecting between the thyroid capsule and the true capsule, revealing a normally sized thyroid gland with a poorly demarcated nodule measuring approximately 0.5 cm by 0.5 cm in the left lobe. The pyramidal lobe was mobilized, and the isthmus was transacted slightly to the right. The superior thyroid artery and vein, middle thyroid vein, and inferior pole branches were ligated to ensure the careful preservation of the parathyroid glands. The left recurrent laryngeal nervewas identified and preserved, with post-resection neurostimulation confirming intact electromyographic signals. The bilateral central compartment and pretracheal lymphatic tissues were excised, and frozen section pathology revealed reactive lymphoid hyperplasia and thymic remnants in the central nodes. Final paraffin-embedded histopathology confirmed a 0.3 cm classical variant of papillary thyroid carcinoma in the left lobe and isthmus, with negative surgical margins and no nodal metastasis (pT1aN0).

**Figure 1.**
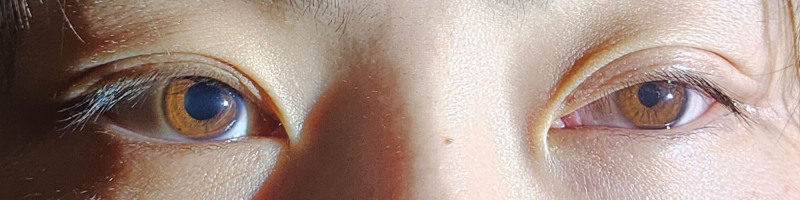
Ocular examination was performed under scotopic conditions with illumination from an inferolateral angle. In primary gaze, left ptosis and miosis were observed. The pupillary diameter was 4.9 mm in the right eye (OD) and 2.8 mm in the left eye (OS). The margin of the right upper eyelid was approximately 2 mm from the corneal limbus. In contrast, the left upper eyelid margin completely covered the superior pupillary border. These findings in the left eye are consistent with Horner syndrome. OD = oculus dexter (right eye), OS = oculus sinister (left eye).

**Figure 2.**
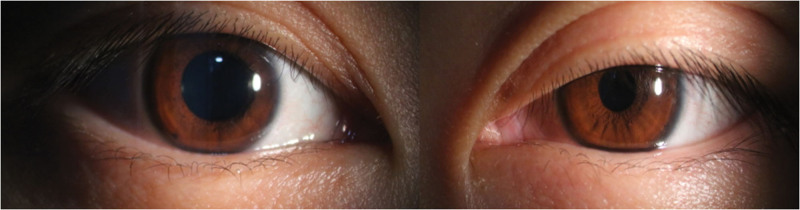
Dark-adapted anterior segment imaging (slit-lamp diffuse illumination with ground glass filter) demonstrated left eyelid ptosis and pupillary miosis. These findings are consistent with Horner syndrome in the left eye.

On the first postoperative day, the patient developed left-sided ptosis, miosis, and mild anhidrosis, which persisted throughout follow-up., raising the suspicion of left-sided HS. Three days later, the patient was discharged and presented to our department for evaluation. Physical examination revealed anhidrosis of the left side of the face after exercise. Ophthalmic examination revealed a visual acuity of 20/20 in both eyes, with intraocular pressures of 16 mm Hg in the right eye and 15 mm Hg in the left eye. Both eyes exhibited orthophoria in primary gaze with full ocular motility. Mild left ptosis was observed, with the right upper eyelid margin covering approximately 2 mm of the superior corneal limbus in the primary gaze, while the left upper eyelid margin was positioned at the superior pupillary border (Fig. 1). After 30 s of dark adaptation in an ophthalmic darkroom, pupil diameters were approximately 4.9 mm in the right eye and 2.8 mm in the left eye (Fig. 2). Both pupils were round and demonstrated prompt direct and consensual light reflexes, with no relative afferent pupillary defectdetected (RAPD). No abnormalities were noted in the anterior segments or fundi of either eye. The results of the remaining neurological examinations were unremarkable.

**Figure 3.**
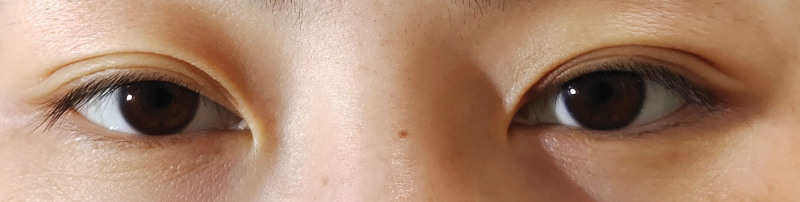
One drop of 0.2% brimonidine tartrate was instilled bilaterally. Examination at 45 minutes post-instillation revealed symmetrical palpebral fissures in primary gaze. The upper eyelids bilaterally covered the superior corneal limbus by approximately 2 mm. Ocular photograph was obtained Under ambient overhead lighting in the ophthalmology examination room. This reversal of anisocoria and ptosis confirms the diagnosis of Horner syndrome in the left eye.

At 45 minutes after instillation of 0.2% brimonidine tartrate, primary gaze examination revealed symmetric palpebral fissures with resolution of the left upper eyelid ptosis (Fig. 3). Based on the patient’s surgical history, ocular symptoms, and clinical signs, pharmacological testing confirmed a diagnosis of preganglionic HS in the left eye. A summary of the diagnostic neuro-ophthalmic evaluation is presented in Table 1 and a detailed timeline outlining the clinical course in Table 2.

**Table 1:** Summary of diagnostic neuro-ophthalmic evaluations.

Evaluation Test	Method	Results	Interpretation
Darkroom pupillometry	Pupil diameter measured after 30s of dark adaptation with inferior flashlight illumination.	OD: 4.9 mm OS: 2.8 mm	Anisocoria greater in dark, consistent with Horner syndrome (impaired dilation on left).
Ptosis quantification	Measurement of upper eyelid margin position relative to corneal limbus in primary gaze.	OD: ~2 mm below the limbus OS: At superior pupillary border	Mild left upper eyelid ptosis.
Brimonidine test	Instillation of 0.2% brimonidine tartrate drops bilaterally; assessment after 45 minutes.	Pretest: Left ptosis and miosis present. Posttest: Symmetric palpebral fissures; ptosis resolved.	Positive test, confirming the diagnosis of Horner syndrome.
Anhidrosis assessment	Patient reporting after exercise.	Anhidrosis on the left side of the face.	Suggests the lesion is proximal to the superior cervical ganglion (preganglionic).

OD = oculus dexter (right eye), OS = oculus sinister (left eye).

**Table 2:** Timeline of clinical course and management

Date/time point	Event/milestone	Description/findings
Preoperative	Thyroid nodule discovery	Asymptomatic nodule found during routine examination.
Preoperative	Fine-needle aspiration cytology	Atypical cells suggestive of papillary thyroid carcinoma; BRAF V600E mutation confirmed.
December 26, 2023	Surgery	Endoscopic extended radical resection (left thyroid lobectomy with isthmusectomy, central compartment lymph node dissection).
Postoperative day 1	Onset of symptoms	Weakness in opening the left eye was observed, suspected left Horner syndrome.
Postoperative day 5	First neuro-ophthalmic evaluation	Presented to the ophthalmology department. Confirmed left miosis, ptosis, and anhidrosis.
Postoperative day 5	Pharmacological test	0.2% Brimonidine tartrate test: positive (ptosis resolved after 45 min).
Postoperative day 5	Treatment initiated	IV methylprednisolone (80 mg/day, 10 d) and oral methylcobalamin (0.5 mg TID, 30 d).
June 2024	6 mo follow-up	Partial improvement in left ptosis and miosis.
April 2025	16 mo follow-up	Near-complete resolution of ptosis and miosis. Patient reported complete resolution of symptoms.

IV = intravenous infusion.

**Figure 4.**
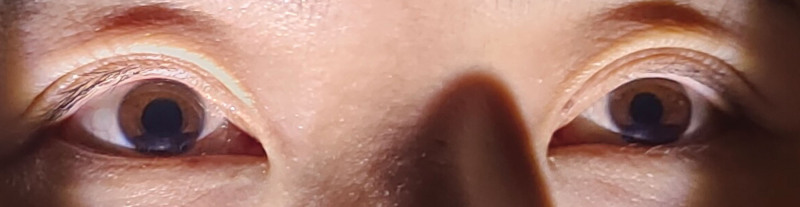
At the 6-mo follow-up, a partial improvement in left upper eyelid ptosis was observed. In primary gaze, the margin of the left upper eyelid was approximately 1 mm lower than that of the right eyelid, with less pronounced left miosis compared to the initial presentation. Pupillometry showing persistent anisocoria consistent with Horner syndrome at 6 mo, highlighting the lack of full recovery and underscoring the need for long-term monitoring.

**Figure 5.**
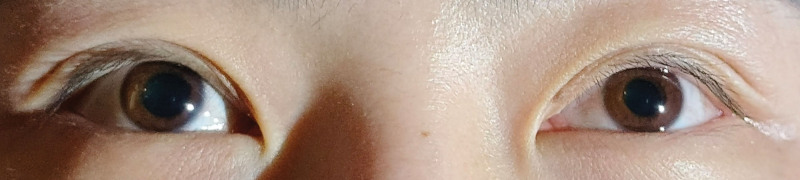
At the 16-mo follow-up, the left upper eyelid ptosis had resolved. In primary gaze, the upper eyelid margins were essentially symmetric bilaterally, and the pupillary size was essentially isocoric. The patient reported a complete resolution of ocular symptoms. These findings indicate clinical resolution of the condition.

Low-dose glucocorticoid therapy was initiated immediately with intravenous methylprednisolone (80 mg once daily) for 10 days. Oral methyl cobalamin (0.5 mg 3 times daily) was administered for 30 days to alleviate the potential cervical sympathetic nerve edema and injury. At the 6-month follow-up, a reduction in the severity of left upper eyelid ptosis and less pronounced left miosis were observed compared to the initial presentation (Fig. 4). At 16 months posttreatment, examination under inferior flashlight illumination in a darkroom revealed nearly symmetrical upper eyelid positions in the primary gaze, with 1 to 2 mm of superior corneal limbus coverage bilaterally. The left pupillary miosis had significantly improved, measuring 4.5 mm compared to 4.9 mm in the right eye (Fig. 5). The patient reported complete resolution of symptoms, including disappearance of right facial anhidrosis, with a full return to normal daily activities and work.

## 3. Discussion

The occurrence of HS following thyroid surgery, including thyroidectomy, thyroidectomy with lateral lymph node dissection, and video-assisted thyroidectomy, has been documented in literature.^[4]^ HS after thyroidectomy may result from direct injury, ischemia, or compression of the sympathetic chain by a postoperative hematoma. The principal cause is often direct mechanical trauma, which can occur from lateral traction during minimally invasive procedures or from injury to the middle cervical ganglion and sympathetic trunk during resection. Additionally, thermal injury from energy devices (e.g., electrocautery, harmonic scalpels) poses a significant risk.^[2]^ Secondary mechanisms include ischemic neural injury due to ligation of the lateral trunk of the inferior thyroid artery, as well as damage resulting from postoperative hematoma, edema, or inflammation along the sympathetic pathway.^[2,5]^ Anatomical variations in the sympathetic chain also increase susceptibility to iatrogenic injury, even during technically sound operations.^[6]^ This case underscores that postoperative HS often arises from a combination of these multifactorial mechanisms.^[7]^ To prevent iatrogenic HS, surgeons should perform meticulous dissection near the cervical sympathetic chain and avoid excessive traction or tissue manipulation.

The definitive diagnosis and localization of HS depend not only on ocular and facial signs but also on pharmacological testing. Traditionally used diagnostic eye drops, such as cocaine, apraclonidine, and hydroxy-amphetamine are not readily available. Therefore, it is essential to identify alternative, easily accessible topical agents for diagnosis. In the case of HS, brimonidine tartrate is regarded as a more selective α-2 adrenergic agonist than apraclonidine because of the denervation super sensitivity of the ocular α-1 receptors. Mechanistically, the affected eyelid in HS demonstrates super sensitivity to the brimonidine tartrate ophthalmic solution. The resultant receptor profile enhances brimonidine super sensitivity, thereby accounting for the observed resolution of ptosis following topical administration.^[8]^

Literature indicates that most patients experience a gradual, progressive amelioration of symptoms, typically beginning within the first 2 to 6 months postoperatively. While many achieve full recovery within this period, a substantial proportion of cases may require up to 1 year for complete resolution.^[9]^ In contrast, our case demonstrated a prolonged recovery, achieving complete resolution of postganglionic HS at 16 months. These findings suggest that some patients experience prolonged recovery periods exceeding 1 year before becoming asymptomatic.

While numerous cases of HS following thyroid surgery have been documented, most lack high-quality ocular photographs obtained under standardized darkroom conditions, a critical diagnostic setting in which pupillary asymmetry (anisocoria) becomes most apparent. This typically manifests as significant dilation of the unaffected pupil with minimal size variation in the affected eye. For suspected post-thyroidectomy HS cases, we strongly recommend the following: meticulous pupillary evaluation by ophthalmologists under darkroom conditions using inferior light illumination to minimize measurement artifacts; bilateral pupillary photographs should be obtained after dark adaptation using inferior flashlight illumination to reduce interference with pupillary size, followed by high-resolution ocular imaging using slit-lamp diffuse illumination with frosted glass filtration to document the anterior segment anatomy; and confirmatory pharmacological testing with brimonidine tartrate ophthalmic solution or alternative ophthalmic agents, and when feasible, precise mapping of the affected anhidrotic area should be performed, potentially including Minor’s starch-iodine test for objective confirmation. Our longitudinal follow-up indicated potential symptom resolution more than 1 year postoperatively, providing valuable prognostic insights for surgeons managing similar cases.

## Acknowledgments

The authors would like to thank the patient featured in this case report.

## Author contributions

**Data curation:** Bo Wang.

**Formal analysis:** Shan-Xue Liu.

**Investigation:** Xiao-Ming Li.

**Resources:** Song Yang.

**Supervision:** Xiao-Ming Li.

**Writing – original draft:** Xiao-Ming Li, Bo Yang.

**Writing – review & editing:** Xiao-Ming Li, Bo Yang.
